# Surgical Outcome of Two-Level Transforaminal Percutaneous Endoscopic Lumbar Discectomy for Far-Migrated Disc Herniation

**DOI:** 10.1155/2016/4924013

**Published:** 2016-12-14

**Authors:** Xinbo Wu, Guoxin Fan, Xin Gu, Xiaofei Guan, Shisheng He

**Affiliations:** ^1^Department of Orthopedic, Tenth People's Hospital, Tongji University, Shanghai 200072, China; ^2^Department of Orthopedic, Tongji Hospital, Tongji University, Shanghai 200072, China

## Abstract

*Objective.* To describe the two-level percutaneous endoscopic lumbar discectomy (PELD) technique in transforaminal approach for highly migrated disc herniation and investigate its clinical outcomes.* Methods.* A total of 22 consecutive patients with highly migrated lumbar disc herniation were enrolled for the study from June 2012 to February 2014.* Results.* There were 12 males and 10 females, with a mean age of 41.1 (range 23–67) years. The mean follow-up period was 18.05 (range 14–33) months. According to the modified MacNab criteria, the clinical outcome at the final follow-up was excellent in 14, good in 6, and fair in 2 patients and the satisfactory rate (excellent and good) was 90.9%. The improvements in VAS and ODI were statistically significant. One patient had recurrent herniation in 18 months after the first surgery and underwent open discectomy. One patient showed symptoms of postoperative dysesthesia (POD), but the POD symptom was transient and partial remission was achieved in two months after conservative treatment.* Conclusion.* Two-level PELD in transforaminal approach can be a safe and effective procedure for highly migrated disc herniation.

## 1. Introduction

Since Kambin [1] introduced the concept of indirect decompression of the spinal canal via posterolateral approach in 1973 and Hijikata [2] described the first percutaneous discectomy in 1975, percutaneous endoscopic lumbar discectomy (PELD) has become a popular surgical option for the management of lumbar disc herniations [3–5]. However, the indications for PELD have been limited mostly to nonmigrated or low-migrated herniations due to inadequate exposure and inability to reach and grasp migrated fragments [6, 7]. Therefore, open surgery was recommended for highly migrated herniations due to a high failure rate of PELD [8, 9].

Recently, many surgeons have developed various novel techniques and instruments that extended the indications of PELD for highly migrated lumbar disc herniations. Choi et al. [7], who used foraminotomy technique to enlarge the foramen by undercutting the ventral part of superior-facet and upper border of inferior pedicle, reported that the foraminotomy was a safe and effective procedure for soft migrated herniations. Although many studies have demonstrated the feasibility through the foraminotomy or contralateral transforaminal approach, the failure rate was high, ranging from 5 to 22% [7, 9–11]. Therefore, the application of these techniques is restricted. Herein, we introduced a two-level PELD technique for highly migrated disc herniations [12]. The purpose of this study was to describe the two-level PELD technique and further reviewed its clinical outcomes.

## 2. Materials and Methods

### 2.1. Study Population

The study was approved by our Institutional Review Board, and all patients were provided with informed consent. We retrospectively reviewed the medical records of 22 patients with buttock and leg pain due to far-migrated disc herniation who underwent two-level PELD by one surgeon (H.S.S) in our department between June 2012 and February 2014. Baseline characteristics for clinical information including age, gender, conservative treatment time, and follow-up were collected. Routine lumbar radiographs along with computed tomography (CT) and magnetic resonance imaging (MRI) scans were conducted to confirm the exact nature and level of pathology. Immediate postoperative MRI was done to ensure successful removal of the herniated remnants. All patients have been followed up without lost via phone or outpatient recheck.

### 2.2. Highly Migrated Disc Herniation

Herniations either above the endplate level of the upper body or below the endplate level of the lower body were called as migrated herniations. According to Lee et al. [9] reports, disc migration was classified into four zones depending on the direction and distance from the disc space: far-upward: from the inferior margin of upper pedicle to 3 mm below the inferior margin of upper pedicle; near-upward: from 3 mm below the inferior margin of upper pedicle to the inferior margin of upper vertebral body; near-downward: from the superior margin of lower vertebral body to the center of lower pedicle; far-downward: from the center to the inferior margin of lower pedicle.

### 2.3. Inclusion and Exclusion Criteria

Inclusion criteria include (1) patients complaining with low back and lower limb pain or numbness and motor weakness due to migrated disc; (2) those with positive straight-leg raising test (<60°) and positive augmentation test, as well as hypoesthesia and decreased muscle force of legs; (3) symptoms corresponding with preoperative MRI and CT scans; (4) unsuccessful conservative treatment for at least 3 months; (5) far-downward and far-upward migrated disc herniation confirmed by MRI or CT.

Exclusion criteria include (1) central stenosis or lateral recess stenosis confirmed by MRI and CT; (2) evident disc calcification confirmed by CT; (3) L1/2 or L2/3 disc herniations or downmigrated herniations at L5-S1 level and upmigrated herniations at L3/4; (4) previous lumbar surgery history for segmental lesions; (5) patients with severe mental illness; (6) near-upward and near-downward migrated disc herniations.

### 2.4. Surgical Technique

**Figure 1:**
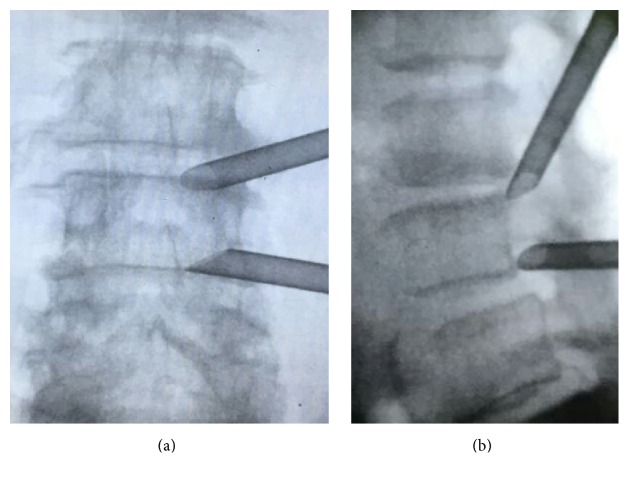
Two working channels placed into the intervertebral foramen at L3/4 and L4/5 level. (a) Anteroposterior fluoroscopy; (b) lateral fluoroscopy.

The two-channel technique would be fully described with the highly downmigrated disc herniations at L3/4 as an example. The patient got prone on the operating table and G-arm fluoroscopy (biplanar 500 mobile biplane fluoroscopy system) was used to confirm the target segment. Prior to the surgery, the patients were informed with all the steps of the procedure. We kept communicating with the patients during the entire surgical procedure. We used self-developed surface locator to conduct preoperative localization to confirm lumbar spinous process, L3, L4, and L5 pedicles, puncture target, and intervertebral space. We also marked the projection of intervertebral foramen on the skin. The surgical puncture point was 10 cm from the midline for L3/4 segment and 11–14 cm from midline for L4/5 segment. Routine disinfection and shop towels were conducted. Lidocaine (1%) was used to conduct the local anesthesia through the puncture pathway with L3/4 free disc as a target. An 18 G needle was inserted into L3/4 intervertebral foramen. Anteroposterior fluoroscopy confirmed the needle positioned on the edge connections pedicle. Lateral fluoroscopy confirmed the needle positioned above the vertebral foramen. The working channel was then placed into the intervertebral foramen, and intraoperative fluoroscopy displayed working channel entirely and diagonally placed on the spinal canal (Figures 1(a) and 1(b)). Endoscopy was placed and yellow ligament was isolated to reveal the top free nucleus pulposus and removed the free nucleus pulposus. To prevent the free nucleus shifted away, the L3/4 working channel was remains.

Lidocaine (1%) was used to conduct the local anesthesia through the puncture pathway. An 18 G needle was localized to L4/5 intervertebral foramen under fluoroscopy, followed by working channels slanted downwards into the foramen. Intraoperative fluoroscopy was used to confirm working channel entirely placed in the spinal canal (Figures 1(a) and 1(b)). The endoscopy was then placed and the prominent free residual nucleus pulposus could be seen located below the L4 nerve root. Curved forceps bit the herniated nucleus pulposus tissue, until no L4 nerve root compressions.

After debriding the L4/5 intervertebral space, we reenter the L3/4 working channel check L4 vertebral posterior. Residual free nucleus remained below the pedicle and then was removed by curved forceps. Finally, we reconfirmed no further remnants alternately via the two channels and flushed the disc space with saline containing gentamicin. The incision was sutured after the working channel was removed.

### 2.5. Observational Parameters

Operation time, hospital stay, postoperative complications were recorded and analyzed. Clinical follow-ups were taken at 3rd month, 12th month, and final follow-up after the surgery. The intensity of pain was measured by the visual analog scale (VAS), ranging from no pain (point 0) to worst pain imaginable (point 10). Patients were assessed functionally on the basis of Oswestry disability index (ODI). The clinical outcome was assessed by an independent surgeon (G.X) using the MacNab criteria [13]. It was defined as excellent outcome as there is no pain and no limitation of normal life; good outcome as there is occasional pain or paresthesia, but no need for medication and no limitation of normal life; fair outcome as pain is somewhat improved but needs medication and some limitation of normal life; poor outcome as no improvement or worsening and additional operation is needed due to incomplete decompression. Excellent and good outcomes were defined as clinically satisfactory.

### 2.6. Statistical Analysis

The Wilcoxon rank sum test and paired *t* test were used to compare the differences of each parameter between preoperative and postoperative VAS and ODI scores. All analyses were performed using SPSS 20.0 (IBM corporation, USA). The result was considered statistically significant if the probability value was less than 0.05.

## 3. Results

There were 12 males and 10 females, with a mean age of 41.1 (range 23–67) years. The mean follow-up period was 18.05 (range 14–33) months (Table 1). The L4-5 disc was the most commonly herniated level (12 cases, 54.5%) followed by L3-4 (6 cases, 27.3%) and L5-S1 (4 cases, 18.2%). There were 14 cases (L4-5 eight cases and L3-4 six cases) of downmigrated herniations and 8 cases (L4-5 four cases, L5-S1 four cases) of upmigrated herniations (Table 2).

According to the modified MacNab criteria, the clinical outcome at final follow-up was excellent in 9 patients, good in 4, and fair in 1 in the downmigrated group and excellent in 5 patients, good in 2, and fair in 1 in the upmigrated group. There was no significant difference between upmigrated group and downmigrated group (*P* = 0.61).

**Figure 2:**
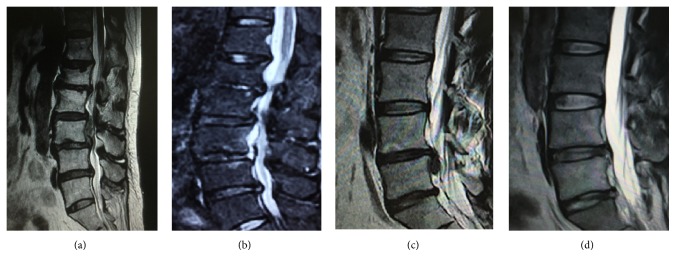
Preoperative and postoperative imaging examination. (a) Preoperative magnetic resonance imaging (MRI) revealed L4/5 disc prolapse with nucleus shifting upward to L3/4 intervertebral space. (b) Postoperative MRI examination revealed clean removal of the nucleus pulposus, with no compression of the L4/5 nerve root. (c) Preoperative magnetic resonance imaging (MRI) revealed L4/5 disc prolapse with nucleus shifting downward to L5 vertebral posterior. (d) Postoperative MRI examination revealed clean removal of the nucleus pulposus, with no compression of the L4/5 nerve root.

Other clinical outcomes were demonstrated in Table 3. The operation time was (88.86 ± 8.0) min. There were significant differences in VAS of back pain and leg pain between before operation and 3 months, 12 months, or final follow-up after operation (*P* < 0.01). Moreover, there were significant differences in ODI between before operation and 3 months, 12 months, or final follow-up after operation (*P* < 0.01). As for complications, 1 patient had recurrent herniation in 18 months after the first surgery and underwent open discectomy. One patient showed symptoms of postoperative dysesthesia (POD); however, POD symptoms were transient and the patient with POD achieved partial remission in two months after conservative treatment. There were no cases of cerebrospinal fluid leak or infections. The migrated disc was completely removed and confirmed by MRI after operation in all patients (Figures 2(a)–2(d)).

## 4. Discussion

PELD in transforaminal approach for highly migrated disc herniation is still challenging due to the limited view and accessibility to the target fragment [9, 14]. Therefore, open surgery was recommended for highly migrated herniations [8]. However, the migrated disc may be separated into multiple fragments. Open surgical approach may need to remove the lamina especially in the region of interarticularis and facets, which may lead to iatrogenic instability and increasing postoperative morbidity [7, 15]. However, compared with the conventional open surgery, PELD has lots of merits such as normal paraspinal structures preservation, minimal postoperative pain, lower risk of postoperative epidural scar formation, and iatrogenic instability [16–23]. Therefore, PELD is a preferred choice for selected patients.

With the development of instruments and technique in the past decade, the indication of PELD was constantly expanding. Many surgeons have reported some new technique for highly migrated disc herniation and achieved favorable clinical outcomes.

Ahn et al. [24] investigated the feasibility of standard PELD with navigable instruments to remove highly migrated disc. Yeom and Choi [25] introduced PELD in a contralateral transforaminal approach for distally migrated disc herniation and the clinical results were excellent in ten patients (10/12) and good in two (2/12) according to MacNab criteria. Choi et al. [7], who used foraminoplastic technique to enlarge the foramen by undercut ventral part of superior-facet and upper border of inferior pedicle, reported that the foraminoplastic-PELD is a safe and effective procedure for surgical treatment of soft migrated herniations. Kim et al. [10] also showed a similar foraminoplasty technique for highly downmigrated disc herniations with favorable clinical outcome in 94% of the patients (50/53). Similarly, Ying et al. [26] conducted foraminoplastic-PELD via upper border of inferior pedicle to remove downmigrated herniations. Kim et al. [14] introduced percutaneous endoscopic interlaminar discectomy for highly migrated disc herniation and the clinical results were excellent in twelve patients (12/18), good in three (3/18), fair in two (2/18), and poor in one (1/18). Similarly, Du et al. [27] also reported the outcomes of percutaneous endoscopic lumbar discectomy via a translaminar approach, especially for soft, highly downmigrated lumbar disc herniation. In our study, we introduced two-level PELD for highly migrated disc herniation through two directions and achieved favorable outcomes. The preoperative VAS of back or leg pain was 7.82 ± 0.96 and 8.59 ± 1.05, which significantly decreased to 2.91 ± 0.61, 2.73 ± 0.46; 2.00 ± 0.54, 1.77 ± 0.69, and 1.14 ± 0.71; 0.95 ± 0.72 at three months, twelve months, and final follow-up. The preoperative ODI were 71.18 ± 7.90, which significantly decreased to 36.55 ± 5.17, 23.36 ± 5.25, and 16.91 ± 4.13 at three months, twelve months, and final follow-up. The improvement in VAS and ODI was statistically significant. According to the MacNab criteria, the clinical results were excellent in 14 patients, good in 6, and fair in two; the satisfactory results (excellent and good) were 90.9%. Therefore, the two-level PELD technique can achieve favorable outcomes.

Although those techniques were excellent and result of the operation was more outstanding compared to the first report by Lee et al. [8], these techniques might have potential risk of disc residues due to the problems like inadequate exposure and inability to reach and grasp herniated fragments [6]. According to the study by Choi et al. [7], 3 of the 59 patients (about 5%) failed to relieve symptoms due to the remnant disc material and remnant disc material was present in 13% (7/53) in the study by Kim et al. [10]. The failure may be due to the characteristic of highly migrated disc herniation. The highly migrated discs were sometimes multifragmented and easily snapped off during pulling of the disc material [9]. Multifragmented disc material was observed in 11 of 18 (about 61%) patients in the study by Kim et al. [14]. Therefore, those fragmented herniations could not be completely removed just by grasping the proximal part of the herniation. Besides, there was some small migrate disc located in pedicle and we could not see it through the endoscope due to anatomic barriers. Those problems were the major causes of postoperative remnant disc materials. In this study, we used two working channels at two levels to confirm whether there was remnant disc and the migrated disc was completely removed and confirmed by magnetic resonance imaging after operation in all patients. The advantage of this setting was that when the debris discs shifted away, we could remove them from the other channel. Therefore, this procedure would reduce the incidence of postoperative residual disc.

## 5. Conclusion

This study demonstrates that two-level PELD in transforaminal approach can be a safe and effective procedure for highly migrated disc herniation. It can be a viable alternative to conventional open surgery because direct approach to the migrated herniations is feasible with this technique.

## Figures and Tables

**Table 1:** Demographic characteristics (*N* = 22).

Variables	Male (*n* = 12)	Female (*n* = 10)	Total
Age (years)	41.2 ± 9.6	41.0 ± 12.7	41.1 ± 10.8
BMI	24.8 ± 2.1	25.4 ± 2.1	24.8 ± 2.1
Lesions in the segments			
L3-L4	4	2	6
L4-L5	7	5	12
L5-S1	3	1	4
Conservative time (months)	8.0 ± 3.6	6.3 ± 1.8	7.5 ± 3.2
Follow-up time (months)	18.8 ± 5.0	17.2 ± 2.5	18.05 ± 4.0
Hospital stay (days)	1.75 ± 0.75	1.60 ± 0.84	1.68 ± 0.78

**Table 2:** The distribution type of nucleus pulposus migration.

Variables	Upward migrated disc herniations	Downward migrated disc herniations
L5/S1	4	0
L4/5	4	8
L3/4	0	6

**Table 3:** VAS back score, VAS leg score, and ODI score in each follow-up time.

Variables	Before operation	Three months after the operation	Twelve months after the operation	Final follow-up
VAS back score	7.82 ± 0.96	2.91 ± 0.61^#^	2.00 ± 0.54^&^	1.14 ± 0.71^*※*^
VAS leg score	8.59 ± 1.05	2.73 ± 0.46^#^	1.77 ± 0.69^&^	0.95 ± 0.72^*※*^
ODI score (%)	71.18 ± 7.90	36.55 ± 5.17^#^	23.36 ± 5.25^&^	16.91 ± 4.13^*※*^

VAS: visual analog scale; ODI: Oswestry disability index.

^#^Compared with preoperative score.

^&^Compared with preoperative score.

^*※*^Compared with preoperative score.

## References

[1] Kambin P., Zhou L. (1997). Arthroscopic discectomy of the lumbar spine. *Clinical Orthopaedics and Related Research*.

[2] Hijikata S. (1989). Percutaneous nucleotomy. A new concept technique and 12 years' experience. *Clinical Orthopaedics and Related Research*.

[3] Khashan M., Lidar Z., Salame K. (2016). Minimally invasive spinal decompression in patients older than 75 years of age: perioperative risks, complications, and clinical outcomes compared with patients younger than 45 years of age. *World Neurosurgery*.

[4] Gadjradj P. S., Van Tulder M. W., Dirven C. M., Peul W. C., Harhangi B. S. (2016). Clinical outcomes after percutaneous transforaminal endoscopic discectomy for lumbar disc herniation: a prospective case series. *Neurosurgical Focus*.

[5] Choi K. C., Kim J. S., Park C. K. (2016). Percutaneous endoscopic lumbar discectomy as an alternative to open lumbar microdiscectomy for large lumbar disc herniation. *Pain Physician*.

[6] Schaffer J. L., Kambin P. (1991). Percutaneous posterolateral lumbar discectomy and decompression with a 6.9-millimeter cannula: analysis of operative failures and complications. *Journal of Bone and Joint Surgery—Series A*.

[7] Choi G., Lee S.-H., Lokhande P. (2008). Percutaneous endoscopic approach for highly migrated intracanal disc herniations by foraminoplastic technique using rigid working channel endoscope. *Spine*.

[8] Lee S.-H., Kang B. U., Ahn Y. (2006). Operative failure of percutaneous endoscopic lumbar discectomy: a radiologic analysis of 55 cases. *SPINE*.

[9] Lee S., Kim S.-K., Lee S.-H. (2007). Percutaneous endoscopic lumbar discectomy for migrated disc herniation: classification of disc migration and surgical approaches. *European Spine Journal*.

[10] Kim H. S., Ju C. I., Kim S. W., Kim J. G. (2009). Endoscopic transforaminal suprapedicular approach in high grade inferior migrated lumbar disc herniation. *Journal of Korean Neurosurgical Society*.

[11] Choi G., Prada N., Modi H. N., Vasavada N. B., Kim J.-S., Lee S.-H. (2010). Percutaneous endoscopic lumbar herniectomy for high-grade down-migrated L4-L5 disc through an L5-S1 interlaminar approach: a technical note. *Minimally Invasive Neurosurgery*.

[12] Wu X., Fan G., Guan X. (2016). Percutaneous endoscopic lumbar discectomy for far-migrated disc herniation through two working channels. *Pain Physician*.

[13] Macnab I. (1971). Negative disc exploration. An analysis of the causes of nerve-root involvement in sixty-eight patients. *The Journal of Bone & Joint Surgery—American Volume*.

[14] Kim C. H., Chung C. K., Woo J. W. (2012). Surgical outcome of percutaneous endoscopic interlaminar lumbar discectomy for highly migrated disc herniation. *Journal of Spinal Disorders and Techniques*.

[15] Osman S. G., Nibu K., Panjabi M. M., Marsolais E. B., Chaudhary R. (1976). Transforaminal and posterior decompressions of the lumbar spine. A comparative study of stability and intervertebral foramen area. *Spine*.

[16] Ahn Y., Lee S.-H., Park W.-M., Lee H.-Y. (2003). Posterolateral percutaneous endoscopic lumbar foraminotomy for L5-S1 foraminal or lateral exit zone stenosis. Technical note. *Journal of Neurosurgery*.

[17] Lee D. Y., Ahn Y., Lee S.-H. (2006). Percutaneous endoscopic lumbar discectomy for adolescent lumbar disc herniation: surgical outcomes in 46 consecutive patients. *Mount Sinai Journal of Medicine*.

[18] Lee S.-H., Chung S.-E., Ahn Y., Kim T.-H., Park J.-Y., Shin S.-W. (2006). Comparative radiologic evaluation of percutaneous endoscopic lumbar discectomy and open microdiscectomy: a matched cohort analysis. *Mount Sinai Journal of Medicine*.

[19] Lee D. Y., Shim C. S., Ahn Y., Choi Y.-G., Kim H. J., Lee S.-H. (2009). Comparison of percutaneous endoscopic lumbar discectomy and open lumbar microdiscectomy for recurrent disc herniation. *Journal of Korean Neurosurgical Society*.

[20] Pan L., Zhang P., Yin Q. (2014). Comparison of tissue damages caused by endoscopic lumbar discectomy and traditional lumbar discectomy: a randomised controlled trial. *International Journal of Surgery*.

[21] Ruetten S., Komp M., Merk H., Godolias G. (2008). Full-endoscopic interlaminar and transforaminal lumbar discectomy versus conventional microsurgical technique: A Prospective, Randomized, Controlled Study. *Spine*.

[22] Ahn S. S., Kim S. H., Kim D. W., Lee B. H. (2016). Comparison of outcomes of percutaneous endoscopic lumbar discectomy and open lumbar microdiscectomy for young adults: a retrospective matched cohort study. *World Neurosurgery*.

[23] Brouwer P. A., Brand R., Van Den Akker-Van Marle M. E. (2015). Percutaneous laser disc decompression versus conventional microdiscectomy in sciatica: a randomized controlled trial. *Spine Journal*.

[24] Ahn Y., Jang I., Kim W. (2016). Transforaminal percutaneous endoscopic lumbar discectomy for very high-grade migrated disc herniation. *Clinical Neurology and Neurosurgery*.

[25] Yeom K.-S., Choi Y.-S. (2011). Full endoscopic contralateral transforaminal discectomy for distally migrated lumbar disc herniation. *Journal of Orthopaedic Science*.

[26] Ying J., Huang K., Zhu M. (2016). The effect and feasibility study of transforaminal percutaneous endoscopic lumbar discectomy via superior border of inferior pedicle approach for down-migrated intracanal disc herniations. *Medicine*.

[27] Du J., Tang X., Jing X., Li N., Wang Y., Zhang X. (2016). Outcomes of percutaneous endoscopic lumbar discectomy via a translaminar approach, especially for soft, highly down-migrated lumbar disc herniation. *International Orthopaedics*.

